# Supplementary material for: scSNViz: visualization and analysis of cell-specific expressed SNVs
Source: Bioinformatics. 2026 Jan 14;42(2):btag023. doi: 10.1093/bioinformatics/btag023 (PMC12866635; doi:10.1093/bioinformatics/btag023)
Supplement: btag023_Supplementary_Data [file btag023_supplementary_data.zip › scSNViz_SupplMethods_010626.pdf]

scSNViz: Visualization and Analysis of Cell-Specific Expressed SNVs

Siera Martinez[1#*], Tushar Sharma[1*], Allen Kim[1], Luke Johnson[1], Vania Ballesteros Prieto[1], Hovhannes Arestakesyan[1], Sunisha Harris[1], Jewel Dias[1], Joseph Golgfrank[2], Nathan Edwards[3], and Anelia Horvath[1#]

#Correspondence
**These authors contributed equally to this work*

[1]McCormick Genomics and Proteomics Center, Department of Biochemistry and Molecular Medicine, School of Medicine and Health Sciences, The George Washington University, Washington, DC 20037, USA
[2]Department of Computer Science, School of Engineering & Applied Science, Georgetown University, Washington,
DC 20057, USA
[3]Department of Biochemistry and Molecular & Cellular Biology, Georgetown University, Washington, DC 20057, USA

Supplementary Materials and Methods

SEQUENCING DATASETS

The sequencing datasets analyzed in this study, covering 28 primary tumor and normal tissues, are publicly accessible through the NCBI Sequence Read Archive (SRA) under accession numbers RRJNA662503 (prostate cancer, pc (Ma *et al.*, 2020)), PRJNA600483 (non-small cell lung carcinoma, (Wang *et al.*, 2019)), PRJNA576876 (cholangiocarcinoma, (Zhang *et al.*, 2020)), and PRJNA573097 (combined neuroblastoma, normal fetal adrenal and normal embryo cohort (Dong *et al.*, 2020)). Sample IDs, tissue type annotations, and basic sequencing metadata are provided in Supplementary Table 1. Comprehensive details on patient demographics, phenotypic data, sample collection, and the 10x Genomics sequencing protocol are available in the original publications. In summary, all samples were prepared using the 10x Genomics 3' UTR v2 or v3 protocol, and sequencing was performed on an Illumina platform with a read length of 150 nucleotides.

SEQUENCING DATA PREPROCESSING

QC, Alignment and Gene Read Counts Estimation

The sequencing datasets from SRA were retrieved and converted into FASTQ format using sratoolkit v3.0.0 (SRA toolkit). Sequencing quality was evaluated with FastQC v0.11.9 (Andrews and others, 2019). For downstream processing, including alignment, read-to-gene assignment, cell barcode resolution, sequencing error correction, and unique molecular identifier (UMI) collapsing, we utilized the STARsolo module of STAR v2.7.10a (Kaminow *et al.*, 2021) with transcript annotations from the GRCh38.79 assembly.

De novo Variant call, Filtering and Annotation Single-cell variant calling was performed using SCExecute v1.3.35 (Edwards *et al.*, 2023) in conjunction with GATK v4.3.0.0 (McKenna *et al.*, 2010) and Strelka2 v2.9.10 (Kim *et al.*, 2018). To ensure high-confidence calls, we applied a unified filtering strategy, retaining only SNVs that passed stringent quality control thresholds in both GATK and Strelka2. Specifically, SNVs were required to meet strict evidence criteria, including a minimum depth of coverage ($DP \geq 5$), at least three unique alternate allele-supporting reads ($AD \geq 3$), and a Phred-scaled quality score ($QUAL \geq 40$). To further minimize technical artifacts, additional filters were applied: Read Position Rank Sum ($RPRS > -2.0$), eliminating variants biased toward read ends,

Mapping Quality Rank Sum (MQRankSum) > -12.5, ensuring comparable mapping quality between reference and alternate alleles, and Base Quality Rank Sum (BQR) > -2.0, prioritizing variants supported by high-quality base calls. SNVs were annotated using the latest version of ANNOVAR (Wang *et al.*, 2010) and the database versions DbSNP 155 (Sherry *et al.*, 2001), COSMIC v101 (Tate *et al.*, 2019), and REDIportal v3.0 (Lo Giudice *et al.*, 2020).

Gene Expression Quantification and Cell Type Annotation

Gene count matrices generated by STARsolo were processed using Seurat v4.0 (Butler *et al.*, 2018), applying initial quality control filtering to remove low-quality cells based on mitochondrial gene expression, genes detected per cell, and UMIs. Filtering criteria were dataset-specific, reflecting differences in quality, cell numbers, and sequencing depth. Following quality control, Seurat's standard pipeline was used to scale and normalize gene expression data, ensuring comparability across cells. Slingshot (Street *et al.*, 2018) was applied for pseudotime analysis, leveraging a non-parametric approach to infer cellular relationships. Cell type annotation was performed using scType (Ianevski *et al.*, 2022), which assigns cell identities based on predefined marker gene sets. Finally, Copy Number Alterations (CNAs) were inferred from scRNA-seq data using CopyKat (Gao *et al.*, 2021).

Variant and Reference Read Counts Estimation of SNVs

The expression of variant and reference alleles for all identified SNVs across individual cells was quantified using SCReadCounts (Prashant *et al.*, 2021). SCReadCounts processes aligned scRNA-seq BAM files to extract and count allele-specific reads, determining the number of reads supporting the variant allele (N_VAR) and reference allele (N_REF) while leveraging UMIs to minimize amplification biases. The expressed Variant Allele Fraction (VAF_RNA = N_VAR / (N_VAR + N_REF)) is then computed for each SNV locus in every cell, including loci covered exclusively by reference reads, enabling detection of monoallelic expression. This approach supports distinction between VAF_RNA = 0 (indicating no variant expression but presence of reference reads) and a complete absence of signal, which reflects either lack of gene expression or insufficient read coverage at the locus due to technical limitations.

Tools and Scripts Availability

All tools and scripts used are either publicly available from original sources or accessible at https://horvathlab.github.io/NGS/.

References

Andrews,S. and others (2019) FastQC: a quality control tool for high throughput sequence data. 2010. *Https://Www.Bioinformatics.Babraham.Ac.Uk/Projects/Fastqc/.*

Butler,A. *et al.* (2018) Integrating single-cell transcriptomic data across different conditions, technologies, and species. *Nat Biotechnol*, **36**.

Dong,R. *et al.* (2020) Single-Cell Characterization of Malignant Phenotypes and Developmental Trajectories of Adrenal Neuroblastoma. *Cancer Cell*, **38**.

Edwards,N. *et al.* (2023) SCExecute: custom cell barcode-stratified analyses of scRNA-seq data. *Bioinformatics*, **39**.

Gao,R. *et al.* (2021) Delineating copy number and clonal substructure in human tumors from single-cell transcriptomes. *Nat Biotechnol*, **39**.

Lo Giudice,C. *et al.* (2020) Investigating RNA editing in deep transcriptome datasets with REDItools and REDIportal. *Nat Protoc*, **15**.

Ianevski,A. *et al.* (2022) Fully-automated and ultra-fast cell-type identification using specific marker combinations from single-cell transcriptomic data. *Nat Commun*, **13**.

Kaminow,B. *et al.* (2021) STARsolo: accurate, fast and versatile mapping/quantification of single-cell and single-nucleus RNA-seq data. *bioRxiv*.

Kim,S. *et al.* (2018) Strelka2: fast and accurate calling of germline and somatic variants. *Nat Methods*, **15**.

Ma,X. *et al.* (2020) Identification of a distinct luminal subgroup diagnosing and stratifying early stage prostate cancer by tissue-based single-cell RNA sequencing. *Mol Cancer*, **19**.
McKenna,A. *et al.* (2010) The genome analysis toolkit: A MapReduce framework for analyzing next-generation DNA sequencing data. *Genome Res*, **20**.
Prashant,N.M. *et al.* (2021) SCReadCounts: estimation of cell-level SNVs expression from scRNA-seq data. *BMC Genomics*, **22**.
Sherry,S.T. *et al.* (2001) DbSNP: The NCBI database of genetic variation. *Nucleic Acids Res*, **29**.
SRA toolkit.
Street,K. *et al.* (2018) Slingshot: Cell lineage and pseudotime inference for single-cell transcriptomics. *BMC Genomics*, **19**.
Tate,J.G. *et al.* (2019) COSMIC: The Catalogue Of Somatic Mutations In Cancer. *Nucleic Acids Res*, **47**.
Wang,K. *et al.* (2010) ANNOVAR: Functional annotation of genetic variants from high-throughput sequencing data. *Nucleic Acids Res*, **38**.
Wang,L. *et al.* (2019) Single-Cell Map of Diverse Immune Phenotypes in the Metastatic Brain Tumor Microenvironment of Non Small Cell Lung Cancer. *bioRxiv*.
Zhang,M. *et al.* (2020) Single-cell transcriptomic architecture and intercellular crosstalk of human intrahepatic cholangiocarcinoma. *J Hepatol*, **73**.
