# Supplementary material for: scSNViz: visualization and analysis of cell-specific expressed SNVs
Source: Bioinformatics. 2026 Jan 14;42(2):btag023. doi: 10.1093/bioinformatics/btag023 (PMC12866635; doi:10.1093/bioinformatics/btag023)
Supplement: btag023_Supplementary_Data [file btag023_supplementary_data.zip › S_Tables1-2_010826.pdf]

Supplementary Table 1.* Datasets utilized in the analyses performed with scSNViz.

##	NCBI IDs		Sample info	Chromium Version	Read Length	N_cells	Reference
	PRJNA	SAMN	tissue				
1	PRJNA662503	SAMN16086830	Prostate cancer	v2	150	1455	*Ma et al., 2020*
2		SAMN16086829		v2	150	2019	
3	PRJNA600483	SAMN13822232	Non-small Cell Lung Carcinoma	v2	150	4074	*Wang et al., 2019*
4		SAMN13822233		v2	150	8381	
5		SAMN13822234		v2	150	7826	
6	PRJNA576876	SAMN13012145	Intrahepatic Cholangiocarcinoma	v2	150	3519	*Zhang et al.,2020*
7		SAMN13012146		v2	150	2453	
8		SAMN13012147		v2	150	3579	
9		SAMN13012148		v2	150	3769	
10		SAMN13012149		v2	150	2738	
11		SAMN13012150		v2	150	4993	
12	PRJNA573097	SAMN12799275	Neuroblastoma	v2	150	4068	*Dong et al., 2020*
13		SAMN12799274		v2	150	5789	
14		SAMN12799273		v2	150	6988	
15		SAMN12799272		v2	150	2997	
16		SAMN12799270		v2	150	6836	
17		SAMN12799269		v2	150	6994	
18		SAMN12799266		v2	150	12488	
19		SAMN12799264		v2	150	16554	
20		SAMN12799263		v2	150	4273	
21		SAMN15453063		v3	150	12441	
22		SAMN15453064		v3	150	7582	
23	PRJNA573097	SAMN12799261	Normal Fetal Adrenal	v2	150	9112	
24		SAMN12799259		v2	150	4916	
25		SAMN12799258		v2	150	26329	
26		SAMN12799257		v2	150	21816	
27	PRJNA573097	SAMN15453062	Normal Embryo	v3	150	19639	
28		SAMN15453069		v3	150	14375	
					**Total**	**228003**	

Supplementary Table 1 Columns:

PRJNA – NCBI BioProject accession number.
SAMN – NCBI BioSample accession number.
Tissue – Tissue or tumor type analyzed.
Chromium Version – Version of the *10x Genomics Chromium* platform used for library preparation and cell barcoding (e.g., v2 = Single Cell 3′ v2 chemistry).
Read Length – Sequencing read length in base pairs.
N _cells – Number of single cells passing quality control.
Reference – Citation number corresponding to the study in the reference list.

Supplementary Table 2.* Set of sceSNVs used to demonstrate set-level visualization features in scSNViz (Figure 1b)

##	SNV	geneList	accession	functionGVS	rsID	Aas	genomesESP	SNV_COS_ID
1	1:1014228_G>A	ISG15	NM_005101.4	missense	1921	SER,ASN	A=5223/G=7783	COSV65106391
2	1:1014274_A>G	ISG15	NM_005101.4	synonymous	8997	VAL	G=10668/A=2336	COSV65106735
3	1:109737079_C>T	GSTM3	NM_000849.5	missense	7483	VAL,ILE	T=3043/C=9963	COSV56661922
4	1:111243307_C>A	CHI3L2	NM_001025197.1	3-prime-UTR	8535	none	unknown	
5	1:111478407_A>G	C1orf162	NM_001300834.2	3-prime-UTR	1054680	none	unknown	
6	1:11750708_T>A	AGTRAP	NM_001040194.1	3-prime-UTR	6540993	none	unknown	
7	1:155087375_A>G	EFNA3	NM_004952.5	3-prime-UTR	2306124	none	unknown	
8	1:161213726_C>T	NDUFS2	NM_001166159.2	synonymous	1136207	ALA	T=1378/C=11628	COSV57154020
9	1:167430725_T>C	CD247	NM_000734.4	3-prime-UTR	947480	none	unknown	
10	1:167430837_T>A	CD247	NM_000734.4	3-prime-UTR	1052231	none	unknown	
11	1:168055440_G>A	DCAF6	NM_001017977.2	intron	16865519	none	unknown	
12	1:192812042_C>G	RGS2	NM_002923.4	3-prime-UTR	4606	none	unknown	
13	1:202005973_G>A	RNPEP,ELF3-AS1	NM_001319182.2	3-prime-UTR	117567081	none	unknown	
14	1:212445997_T>C	NENF	NM_013349.5	synonymous	4804	ASP	C=5623/T=7383	COSV65340955
15	1:22661465_A>G	C1QB	NM_000491.5	3-prime-UTR	10580	none	unknown	
16	1:228494629_C>G	RNF187	NM_001010858.3	3-prime-UTR	0	none	unknown	
17	1:25900804_G>A	STMN1	NM_001145454.3	intron	0	none	unknown	
18	1:77773018_A>G	none	none	intergenic	0	none	unknown	
19	1:77773045_T>C	none	none	intergenic	0	none	unknown	
20	1:77773058_A>G	none	none	intergenic	0	none	unknown	
21	1:944307_T>C	NOC2L,SAMD11	NM_015658.4	3-prime-UTR	2839	none	unknown	COSV58988633
22	10:133362587_G>A	ECHS1	NM_004092.4	3-prime-UTR	4604	none	unknown	
23	10:17237563_G>T	VIM	NM_003380.5	3-prime-UTR	1049341	none	unknown	
24	10:3136580_C>G	PFKP	NM_001242339.1	3-prime-UTR	184205778	none	C=13006	
25	10:3136623_G>C	PFKP	NM_001242339.1	3-prime-UTR	9063	none	C=1569/G=11437	COSV56538751
26	10:3136716_T>C	PFKP	NM_001242339.1	3-prime-UTR	542	none	unknown	COSV56525277
27	10:68342992_C>A	HNRNPH3,RUFY2	NM_001322434.1	3-prime-UTR	3199937	none	unknown	
28	11:16755795_G>A	C11orf58	NM_014267.6	3-prime-UTR	4576801	none	unknown	
29	11:1892147_A>G	LSP1	NM_001013253.2	3-prime-UTR	548195	none	unknown	
30	11:46383776_C>A	MDK	NM_001012333.2	3-prime-UTR	116869512	none	unknown	COSV58994791
31	11:47354897_G>A	SPI1	NM_001080547.2	3-prime-UTR	1057233	none	unknown	
32	11:67585218_A>G	GSTP1	NM_000852.4	missense	1695	ILE,VAL	G=4535/A=8033	COSV66992376
33	11:78079607_T>C	NDUFC2,NDUFC2-KCTD14	NM_001203260.2	synonymous	534418	LEU	C=11218/T=1602	COSV55247502
34	11:93536126_G>A	SMCO4	NM_020179.3	intron	0	none	unknown	
35	12:132704876_G>A	PXMP2	NM_018663.3	3-prime-UTR	10007	none	unknown	
36	12:55725474_C>T	CD63	NM_001257389.1	3-prime-UTR	1037113923	none	unknown	COSV57677305
37	12:55725822_A>G	CD63	NM_001257389.1	synonymous	0	ALA	A=13006	
38	12:55757507_T>C	SARNP	NM_033082.4	3-prime-UTR	7068	none	C=2854/T=10152	COSV60242616
39	12:9598073_A>G	KLRB1	NM_002258.3	missense	1135816	ILE,THR	G=4425/A=8565	COSV57590625
40	14:103519918_C>T	CKB	NM_001362531.2	synonymous	1803283	GLU	T=7592/C=5410	COSV62379683
41	14:105488785_G>A	CRIP1	NM_001311.5	3-prime-UTR	0	none	unknown	
42	14:20782207_T>C	RNASE6	NM_005615.5	3-prime-UTR	7156801	none	unknown	
43	14:35402011_G>A	NFKBIA	NM_020529.3	3-prime-UTR	8904	none	A=5786/G=7220	COSV53754079
44	14:49586377_G>A	RPS29	NM_001030001.4	5-prime-UTR	1282480270	none	G=13006	COSV99817971
45	14:77708130_A>C	SLIRP	NM_001267863.1	synonymous	11159286	ARG	C=12016/A=990	COSV53174819
46	14:98972797_C>T	none	none	intergenic	372490154	none	unknown	
47	15:34341832_A>G	NOP10	NM_018648.4	3-prime-UTR	3063	none	unknown	COSV60995282
48	15:34341923_C>G	NOP10	NM_018648.4	3-prime-UTR	1045238	none	G=2052/C=10946	COSV60995138
49	15:34341937_G>A	NOP10	NM_018648.4	3-prime-UTR	1045204	none	A=1736/G=11262	COSV60995143
50	15:34341938_T>C	NOP10	NM_018648.4	3-prime-UTR	1045194	none	C=1928/T=11070	COSV60995151

Supplementary Table 2.* Set of sceSNVs used to demonstrate set-level visualization features in scSNViz (cont.)

##	SNV	geneList	accession	functionGVS	rsID	Aas	genomesESP	SNV_COS_ID
51	15:44717976_G>A	B2M	NM_004048.3	3-prime-UTR	0	none	unknown	
52	15:48878780_G>A	EID1,SHC4	NM_014335.3	3-prime-UTR	16961791	none	unknown	
53	15:69452821_C>T	RPLP1	NM_001003.3	5-prime-UTR	529326572	none	unknown	
54	15:81308981_A>G	IL16	NM_001172128.2	3-prime-UTR	859	none	unknown	
55	15:88652295_A>G	ISG20	NM_001303233.2	synonymous	1137166	LEU	G=10738/A=2260	COSV60143565
56	15:92898037_G>A	LINC01578	NR_037600.1	non-coding-exo	7743	none	unknown	
57	16:1325262_T>G	UBE2I	NM_003345.5	3-prime-UTR	7302	none	T=4566	
58	16:57275_T>G	SNRNP25	NM_024571.4	3-prime-UTR	1045001	none	unknown	COSV51954838
59	16:84565600_C>T	COTL1	NM_021149.5	3-prime-UTR	0	none	unknown	
60	16:84565720_G>A	COTL1	NM_021149.5	3-prime-UTR	774809134	none	unknown	
61	16:85922438_A>T	IRF8	NM_001363907.1	3-prime-UTR	6638	none	unknown	
62	16:87483044_A>T	ZCCHC14	NM_015144.3	intron	11864816	none	unknown	
63	17:15229806_C>A	PMP22	NM_000304.4	3-prime-UTR	7415	none	unknown	COSV56601929
64	17:5499637_A>G	LOC728392	NM_001162371.3	3-prime-UTR	5862	none	unknown	
65	17:68532637_C>G	PRKAR1A,FAM20A	NM_001276289.1	3-prime-UTR	6958	none	unknown	COSV56837023
66	17:7014384_C>T	RNASEK,RNASEK-C17orf49	NM_001004333.4	3-prime-UTR	7338	none	unknown	COSV52351111
67	17:7014479_T>C	RNASEK,RNASEK-C17orf49	NM_001004333.4	3-prime-UTR	12135	none	unknown	
68	17:7240720_G>A	GABARAP	NM_007278.2	3-prime-UTR	0	none	unknown	
69	18:59158639_T>C	SEC11C	NM_001307941.2	missense	0	PHE,LEU	T=13006	
70	19:10110966_A>G	PPAN,PPAN-P2RY11	NM_001040664.3	missense	11559188	GLN,ARG	G=613/A=12391	
71	19:10115101_A>G	P2RY11,EIF3G,PPAN-P2RY1	NM_001040664.3	3-prime-UTR	7401	none	G=4884/A=8122	COSV53452898
72	19:1038894_C>T	CNN2	NM_001303499.2	3-prime-UTR	1057895	none	unknown	
73	19:1106616_T>C	GPX4	NM_001039847.3	synonymous	713041	LEU	C=7154/T=4930	COSV62321056
74	19:11450807_A>G	PRKCSH	NM_001001329.2	3-prime-UTR	1056893467	none	unknown	
75	19:11553206_G>A	ELOF1	NM_001363673.1	3-prime-UTR	72620552	none	unknown	COSV52947359
76	19:16133516_G>C	RAB8A	NM_005370.5	3-prime-UTR	1043452	none	unknown	
77	19:17320106_G>A	DDA1	NM_024050.6	3-prime-UTR	10259	none	unknown	
78	19:17320166_G>A	DDA1	NM_024050.6	3-prime-UTR	1059767	none	unknown	
79	19:17403117_G>C	BST2	NM_004335.4	3-prime-UTR	13485	none	unknown	
80	19:17788341_G>A	FCHO1	NM_001161357.2	3-prime-UTR	369047298	none	A=1/G=13003	
81	19:19201687_C>G	RFXANK,NR2C2AP	NM_001278727.1	missense	1802498	GLN,GLU	G=14/C=12992	COSV57393414
82	19:2732743_G>A	SLC39A3	NM_144564.5	3-prime-UTR	9160	none	A=1254/G=11714	
83	19:2754794_G>A	SGTA	NM_003021.4	3-prime-UTR	7009	none	unknown	
84	19:2754812_C>T	SGTA	NM_003021.4	3-prime-UTR	13282	none	unknown	
85	19:2754980_T>C	SGTA	NM_003021.4	3-prime-UTR	7008	none	unknown	
86	19:33387291_G>A	PEPD	NM_000285.4	3-prime-UTR	77690463	none	unknown	COSV52287665
87	19:37738477_G>A	ZNF573	NM_001172689.1	3-prime-UTR	1291	none	A=8975/G=4031	COSV59828169
88	19:38878729_T>G	SIRT2	NM_001193286.1	3-prime-UTR	2015	none	unknown	
89	19:38878874_G>A	SIRT2	NM_001193286.1	3-prime-UTR	2241703	none	unknown	
90	19:40765199_T>C	SNRPA	NM_004596.5	3-prime-UTR	13108	none	C=1532/T=11474	
91	19:40796801_C>T	RAB4B,MIA-RAB4B,RAB4B-EG	NM_016154.5	3-prime-UTR	7937	none	unknown	COSV58279921
92	19:4174401_C>G	SIRT6	NM_001193285.3	3-prime-UTR	350846	none	unknown	
93	19:44903416_G>A	TOMM40	NM_001128916.1	3-prime-UTR	10119	none	unknown	
94	19:45475117_A>G	FOSB	NM_001114171.2	3-prime-UTR	1049739	none	unknown	
95	19:45526896_G>A	VASP	NM_003370.4	3-prime-UTR	10995	none	unknown	
96	19:48966669_G>A	FTL	NM_000146.4	synonymous	0	ARG	G=13006	
97	19:48966687_T>G	FTL	NM_000146.4	synonymous	0	ALA	T=13006	
98	19:48966709_G>A	FTL	NM_000146.4	missense	768204975	GLU,LYS	G=13006	COSV53169512
99	19:48966747_G>C	FTL	NM_000146.4	3-prime-UTR	1432695180	none	G=13006	
100	19:48966766_G>C	FTL	NM_000146.4	3-prime-UTR	764013805	none	G=13006	

Supplementary Table 2.* Set of sceSNVs used to demonstrate set-level visualization features in scSNViz (cont.)

##	SNV	geneList	accession	functionGVS	rsID	Aas	genomesESP	SNV_COS_ID
101	19:48966787_C>T	FTL	NM_000146.4	3-prime-UTR	771233352	none	C=7264	
102	19:49447041_C>T	PIH1D1	NM_017916.3	missense	13394	VAL,ILE	T=10532/C=2474	COSV51807272
103	19:50797973_G>A	C19orf48	NM_001290149.1	3-prime-UTR	9991	none	unknown	COSV54517312
104	19:52734417_T>G	ZNF611	NM_001161499.2	intron	707303	none	unknown	
105	19:55643352_G>A	ZNF580,ZNF581	NM_001163423.1	3-prime-UTR	11233	none	unknown	COSV54402651
106	19:58394796_T>C	RPS5	NM_001009.4	3-prime-UTR	2241787	none	C=5824/T=7182	COSV52190570
107	19:58582097_C>T	none	none	intergenic	0	none	unknown	
108	19:58582117_G>T	none	none	intergenic	0	none	unknown	
109	19:7116272_G>C	INSR	NM_000208.4	3-prime-UTR	1051651	none	unknown	
110	19:7647391_A>G	STXBP2	NM_001127396.3	missense	6791	ILE,VAL	G=8655/A=4315	COSV55401668
111	19:8311547_G>A	NDUFA7	NM_005001.5	synonymous	561	PRO	A=1819/G=10649	COSV56846606
112	19:896740_C>G	R3HDM4	NM_138774.4	3-prime-UTR	7969	none	unknown	COSV54122929
113	2:112829856_C>G	IL1B	NM_000576.3	3-prime-UTR	1071676	none	unknown	
114	2:119372544_T>A	DBI	NM_001079862.3	3-prime-UTR	0	none	unknown	
115	2:174720839_T>A	none	none	intergenic	0	none	unknown	
116	2:174720854_G>A	none	none	intergenic	0	none	unknown	
117	2:232480018_G>C	ECEL1	NM_001290787.2	3-prime-UTR	2741278	none	unknown	COSV58811853
118	2:241495187_A>G	STK25	NM_001271977.2	3-prime-UTR	7618	none	unknown	
119	2:241676047_T>C	DTYMK	NM_001165031.2	3-prime-UTR	5860	none	unknown	COSV59870962
120	2:26190948_C>T	HADHA,GAREM2	NM_000182.5	3-prime-UTR	1049987	none	unknown	
121	2:277995_A>G	ACP1	NM_004300.4	3-prime-UTR	6855	none	unknown	
122	2:47160272_A>G	CALM2	NM_001305624.1	3-prime-UTR	0	none	unknown	
123	2:47160299_C>T	CALM2	NM_001305624.1	3-prime-UTR	11551467	none	unknown	
124	2:85581901_G>A	VAMP8	NM_003761.5	3-prime-UTR	0	none	unknown	
125	2:85698725_C>G	GNLY	NM_001302758.2	3-prime-UTR	12845	none	unknown	COSV55702702
126	20:18763739_T>C	DTD1	NM_080820.6	3-prime-UTR	9139	none	unknown	
127	20:24959885_A>G	CST7	NM_003650.4	3-prime-UTR	1056036	none	unknown	
128	20:4024544_C>T	none	none	intergenic	878976490	none	unknown	
129	20:4024575_G>A	none	none	intergenic	6084555	none	unknown	
130	20:4024581_C>T	none	none	intergenic	8122653	none	unknown	
131	20:46016514_T>C	MMP9,SLC12A5-AS1	NM_004994.3	3-prime-UTR	9509	none	unknown	
132	20:5925133_C>A	CHGB	NM_001819.3	3-prime-UTR	2821	none	unknown	
133	20:62387103_C>T	RPS21	NM_001024.4	5-prime-UTR	972475308	none	unknown	
134	21:44886223_G>T	ITGB2	NM_000211.5	3-prime-UTR	1160263	none	unknown	COSV56609771
135	22:19435833_G>T	MRPL40	NM_001318151.2	synonymous	77131689	ARG	T=2/G=13004	
136	22:32532118_T>C	SYN3	NM_001135774.2	intron	10460755	none	unknown	
137	22:41663764_G>T	XRCC6	NM_001288976.2	synonymous	132788	GLY	T=3487/G=9519	COSV63752372
138	22:43162920_A>G	TSPO	NM_000714.6	missense	6971	THR,ALA	G=9431/A=3553	COSV53355454
139	22:43163131_G>T	TSPO	NM_000714.6	3-prime-UTR	6973	none	unknown	
140	22:49963768_C>T	PIM3	NM_001001852.4	3-prime-UTR	13811	none	unknown	
141	22:50525807_G>A	TYMP,SCO2	NM_001113755.3	missense	11479	SER,LEU	A=846/G=11530	COSV52688025
142	22:50526017_A>T	TYMP,SCO2	NM_001113755.3	synonymous	1138404	GLY	unknown	
143	3:129314956_C>A	H1-10	NM_006026.4	3-prime-UTR	0	none	unknown	
144	3:169966810_C>G	SEC62	NM_003262.4	5-prime-UTR	375131407	none	T=1/C=12563	
145	3:46922012_T>C	CCDC12	NM_001277074.1	3-prime-UTR	4587	none	C=9269/T=3737	COSV52765225
146	4:108625210_T>A	RPL34	NM_000995.4	stop-lost	0	stop,LYS	T=12978	
147	4:17486621_G>A	QDPR	NM_000320.3	3-prime-UTR	1031327	none	unknown	
148	4:17486663_T>G	QDPR	NM_000320.3	3-prime-UTR	699460	none	unknown	
149	4:17486723_G>A	QDPR	NM_000320.3	3-prime-UTR	1049601	none	unknown	
150	4:17486728_T>C	QDPR	NM_000320.3	3-prime-UTR	1049600	none	unknown	

Supplementary Table 2.* Set of sceSNVs used to demonstrate set-level visualization features in scSNViz (cont.)

##	SNV	geneList	accession	functionGVS	rsID	Aas	genomesESP	SNV_COS_ID
151	4:6642664_G>A	MRFAP1	NM_001272053.1	3-prime-UTR	1059220	none	unknown	
152	4:87982701_C>T	SPP1	NM_000582.2	synonymous	1126616	ALA	T=3124/C=9882	COSV52951697
153	4:87983190_A>C	SPP1	NM_000582.2	3-prime-UTR	9138	none	unknown	
154	5:10264964_C>T	CCT5	NM_001306153.1	3-prime-UTR	699113	none	unknown	
155	5:177306115_A>G	PRELID1,MXD3	NM_001142935.2	3-prime-UTR	4631	none	G=11724/A=1282	COSV57462840
156	5:177306615_T>C	PRELID1,MXD3	NM_001142935.2	3-prime-UTR	9834	none	C=11645/T=1173	COSV57463085
157	5:35871088_G>A	IL7R	NM_002185.5	missense	1494555	VAL,ILE	A=9700/G=3306	COSV57406117
158	5:53683267_G>A	NDUFS4	NM_001318051.2	3-prime-UTR	567	none	A=5456/G=7550	COSV57019229
159	6:111764035_A>G	FYN	NM_002037.5	intron	6933144	none	unknown	COSV57611981
160	6:122725777_A>G	PKIB	NM_001270393.1	3-prime-UTR	1132635	none	unknown	COSV57794791
161	6:132814568_C>G	none	none	upstream-gene	576858600	none	unknown	
162	6:132814747_A>G	RPS12	NM_001016.4	5-prime-UTR	9483504	none	G=6929/A=6077	COSV57767099
163	6:149811183_A>T	PCMT1	NM_001252049.1	3-prime-UTR	4552	none	unknown	COSV66308362
164	6:159778994_A>C	ACAT2,TCP1	NM_001008897.1	3-prime-UTR	4832	none	C=2316/A=10682	COSV58458988
165	6:26104052_T>C	H4C3	NM_003542.4	synonymous	2229768	ILE	C=2952/T=10054	COSV58090568
166	6:26104289_T>C	H4C3	NM_003542.4	3-prime-UTR	0	none	T=13006	
167	6:26104297_C>A	H4C3	NM_003542.4	3-prime-UTR	1280403614	none	C=13006	
168	6:26104303_C>A	H4C3	NM_003542.4	3-prime-UTR	1205348791	none	C=13006	
169	6:29555869_G>C	UBD	NM_006398.4	3-prime-UTR	444013	none	C=5024/G=3416	
170	6:29555899_C>G	UBD	NM_006398.4	missense	8337	CYS,SER	G=6756/C=1682	
171	6:31268757_G>A	HLA-C	NM_002117.6	3-prime-UTR	35075694	none	unknown	
172	6:31268790_T>C	HLA-C	NM_002117.6	3-prime-UTR	1049281	none	unknown	
173	6:31268866_T>C	HLA-C	NM_002117.6	3-prime-UTR	1130552	none	unknown	
174	6:31268902_T>C	HLA-C	NM_002117.6	3-prime-UTR	1130580	none	unknown	COSV66115208
175	6:31268913_T>G	HLA-C	NM_002117.6	3-prime-UTR	1130592	none	unknown	COSV66119531
176	6:31616832_G>A	AIF1	NM_001318970.2	missense	753881781	GLU,LYS	G=13006	
177	6:31616909_G>A	AIF1	NM_001318970.2	3-prime-UTR	0	none	G=13006	
178	6:31669957_A>T	CSNK2B	NM_001282385.1	3-prime-UTR	5872	none	T=1883/A=6557	COSV65507945
179	6:31730686_C>T	CLIC1	NM_001287593.1	3-prime-UTR	0	none	unknown	
180	6:36602589_T>C	SRSF3	NM_003017.5	3-prime-UTR	7344	none	unknown	COSV59672440
181	6:37483138_T>C	CCDC167	NM_138493.3	3-prime-UTR	10692	none	C=9630/T=3376	COSV64982259
182	6:85678170_G>T	SNHG5	NR_003038.2	non-coding-ex	1059307	none	unknown	
183	6:85678734_A>C	none	none	upstream-gene	1207944590	none	unknown	
184	7:150338437_T>G	RARRES2	NM_002889.4	3-prime-UTR	4721	none	unknown	COSV56231044
185	7:150791528_A>G	TMEM176B	NM_001101311.1	3-prime-UTR	2302479	none	G=8453/A=4553	COSV58439096
186	7:24285390_G>A	NPY	NM_000905.4	synonymous	5573	SER	A=5945/G=7061	COSV54215135
187	7:42937596_G>A	MRPL32	NM_031903.3	3-prime-UTR	631561	none	A=12789/G=213	
188	8:123015388_T>C	DERL1	NM_001134671.2	3-prime-UTR	7159	none	unknown	COSV52364774
189	8:22594175_G>C	PDLIM2	NM_001368120.1	3-prime-UTR	3064	none	unknown	COSV56133534
190	8:32094528_A>T	NRG1,NRG1-IT1	NM_001159995.3	intron	16878794	none	unknown	
191	8:79666159_T>A	STMN2	XM_005251142.2	3-prime-UTR	904037857	none	unknown	
192	9:127451406_G>C	RPL12	NM_000976.4	5-prime-UTR	747333236	none	unknown	
193	9:137273425_T>G	NELFB	NM_015456.5	3-prime-UTR	8281	none	unknown	
194	9:36211830_C>T	CLTA	NM_001076677.3	3-prime-UTR	1053414	none	unknown	COSV54254584
195	9:93121503_T>C	NINJ1	NM_004148.4	3-prime-UTR	7033638	none	unknown	
196	9:93121564_G>A	NINJ1	NM_004148.4	3-prime-UTR	12238760	none	unknown	
197	9:93121676_A>T	NINJ1	NM_004148.4	3-prime-UTR	1127851	none	unknown	
198	X:23786051_C>T	SAT1	NM_002970.3	3-prime-UTR	0	none	unknown	
199	X:23786107_G>A	SAT1	NM_002970.3	3-prime-UTR	0	none	unknown	
200	X:47585586_T>C	SYN1,TIMP1	NM_003254.3	synonymous	4898	PHE	C=4892/T=5671	COSV54483037
201	X:81298369_A>T	SH3BGRL	NM_003022.3	3-prime-UTR	149428539	none	unknown	

**Supplementary Table 2 Columns:
– Variant index number in this table.
SNV – Genomic position and base substitution in the format *chromosome:position_ref>alt* (hg38 coordinates).
geneList – Gene or genes associated with the variant, as annotated by the transcript reference.
accession – NCBI RefSeq transcript accession number corresponding to the annotated gene.
functionGVS – Functional classification of the variant based on predicted consequence (e.g., *missense*, *synonymous*, *3-prime-UTR*, *intron*, *intergenic*).
rsID – dbSNP reference identifier when available.
Aas – Amino-acid substitution (reference and alternate residues) for coding variants, or *none* if the variant is non-coding.
genomesESP – Allele frequency or count information from population databases (*1000 Genomes*, *ESP*), where available; *unknown* indicates absence of data.
SNV_COS_ID – COSMIC variant identifier (COSV), referencing the COSMIC database entry if previously reported.
