# Supplementary figures and images for: scSNViz: visualization and analysis of cell-specific expressed SNVs
Source: Bioinformatics. 2026 Jan 14;42(2):btag023. doi: 10.1093/bioinformatics/btag023 (PMC12866635; doi:10.1093/bioinformatics/btag023)

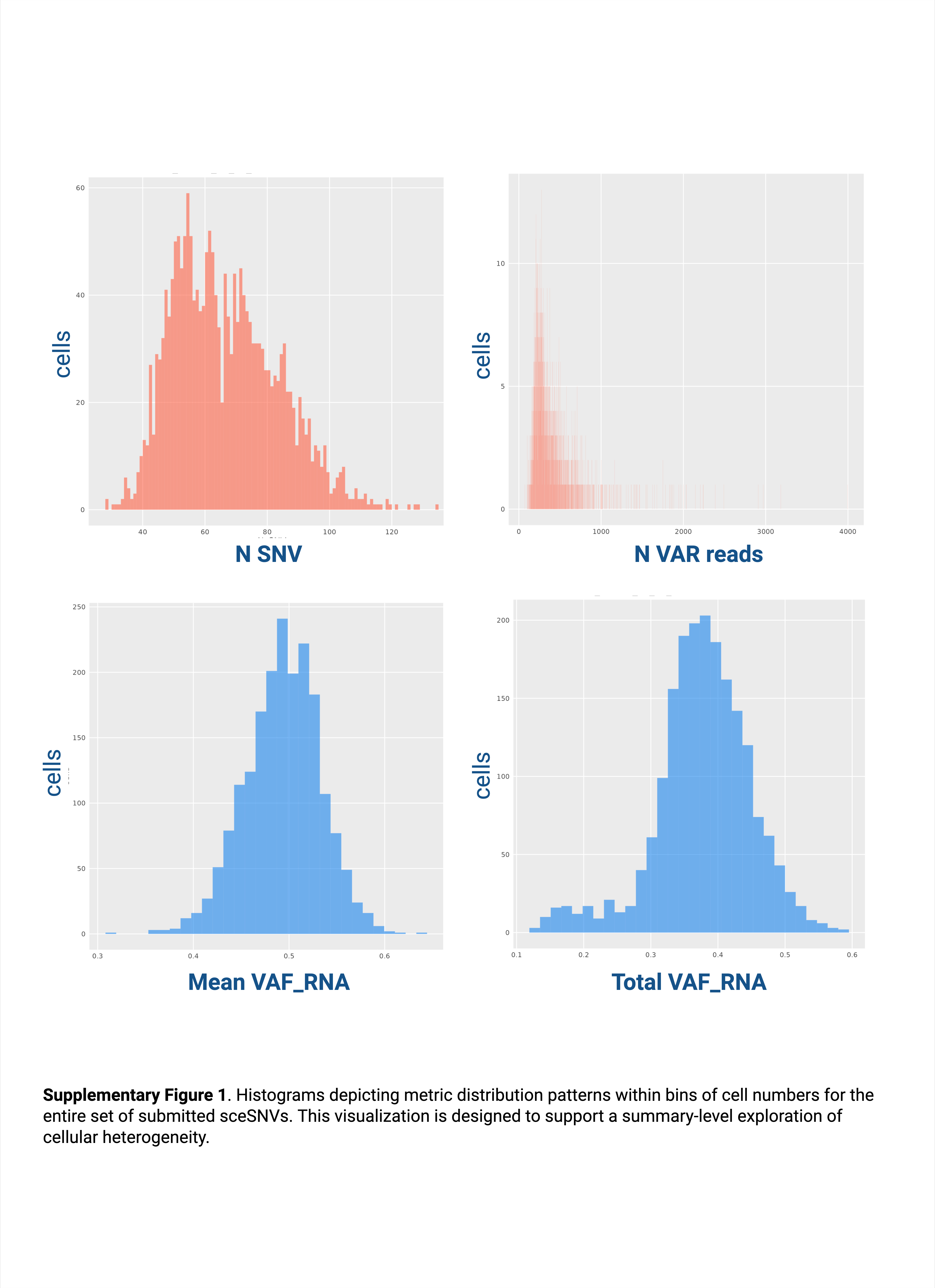

Supplement: btag023_Supplementary_Data [file btag023_supplementary_data.zip › ScSNViz_S_Figure_1.jpg]

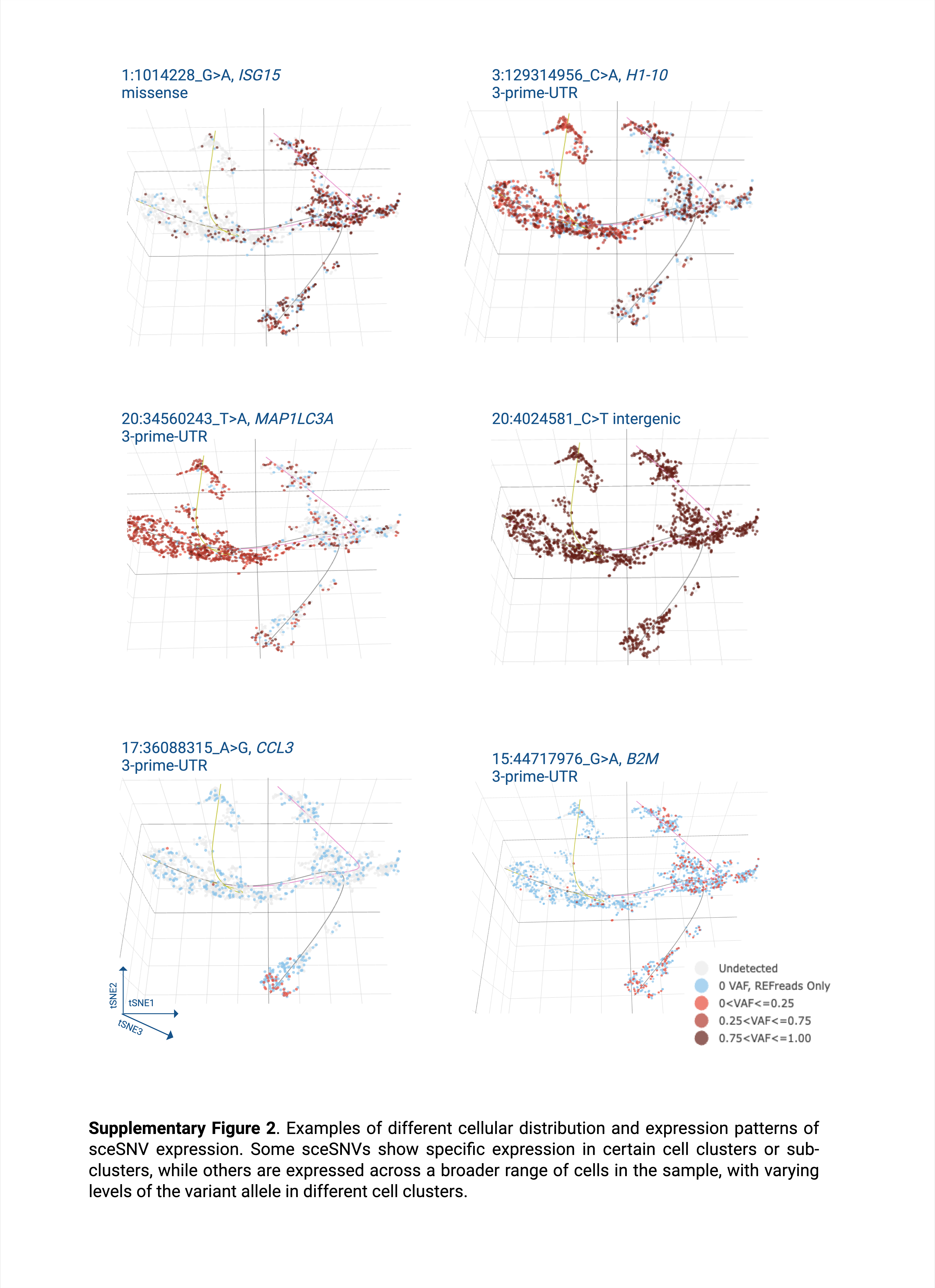

Supplement: btag023_Supplementary_Data [file btag023_supplementary_data.zip › ScSNViz_S_Figure_2.jpg]

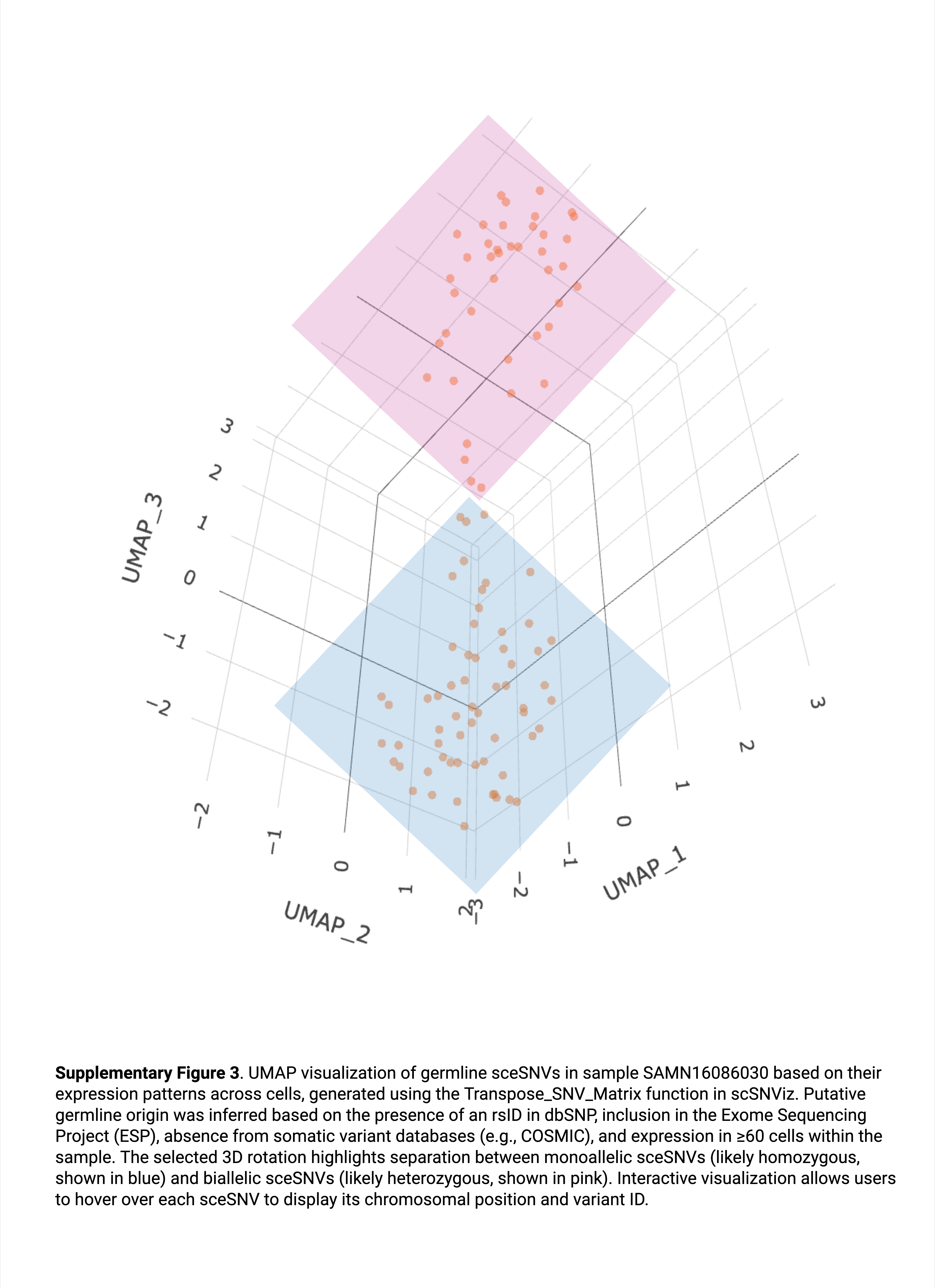

Supplement: btag023_Supplementary_Data [file btag023_supplementary_data.zip › ScSNViz_S_Figure_3.jpg]

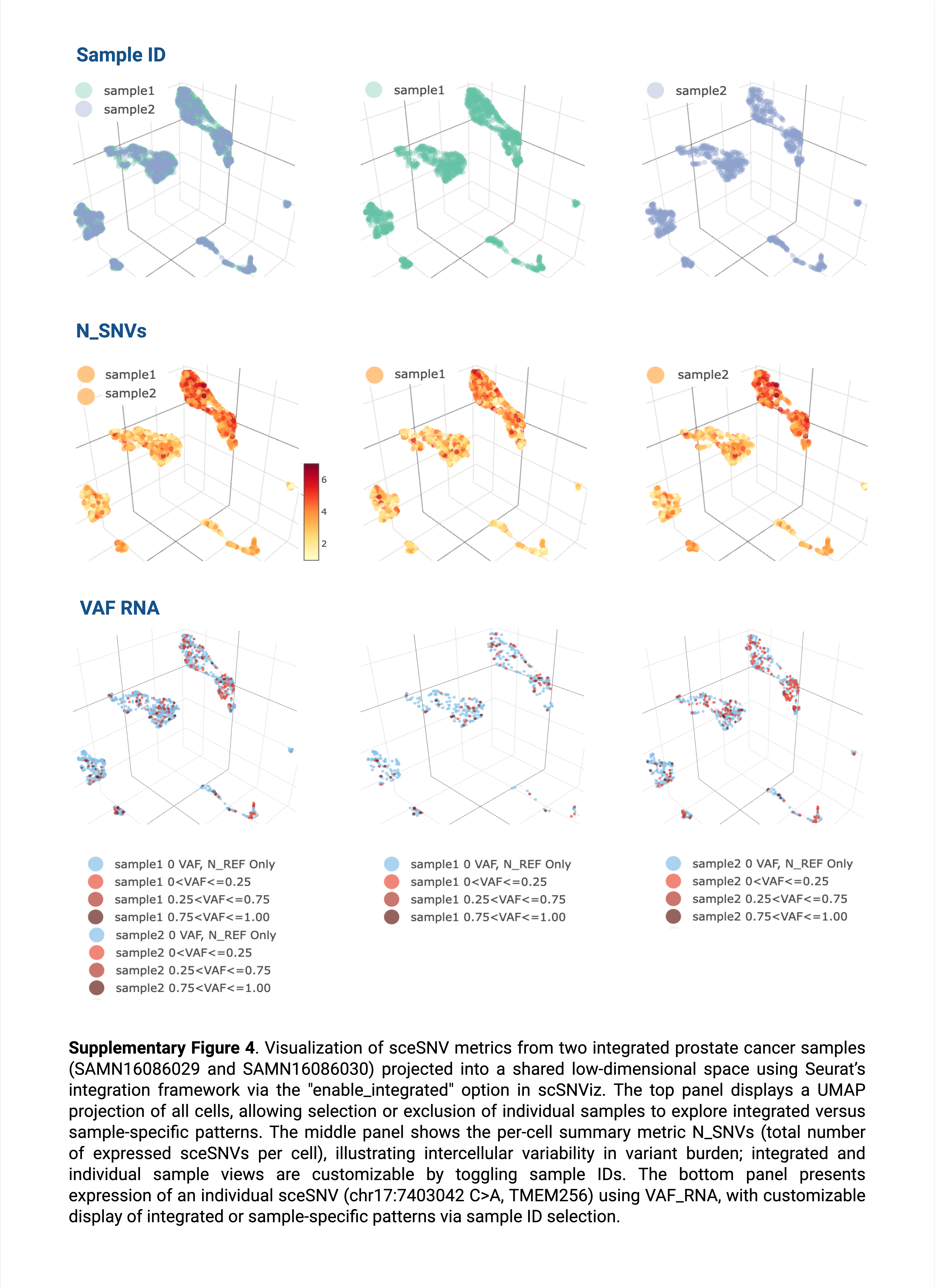

Supplement: btag023_Supplementary_Data [file btag023_supplementary_data.zip › ScSNViz_S_Figure_4.jpg]

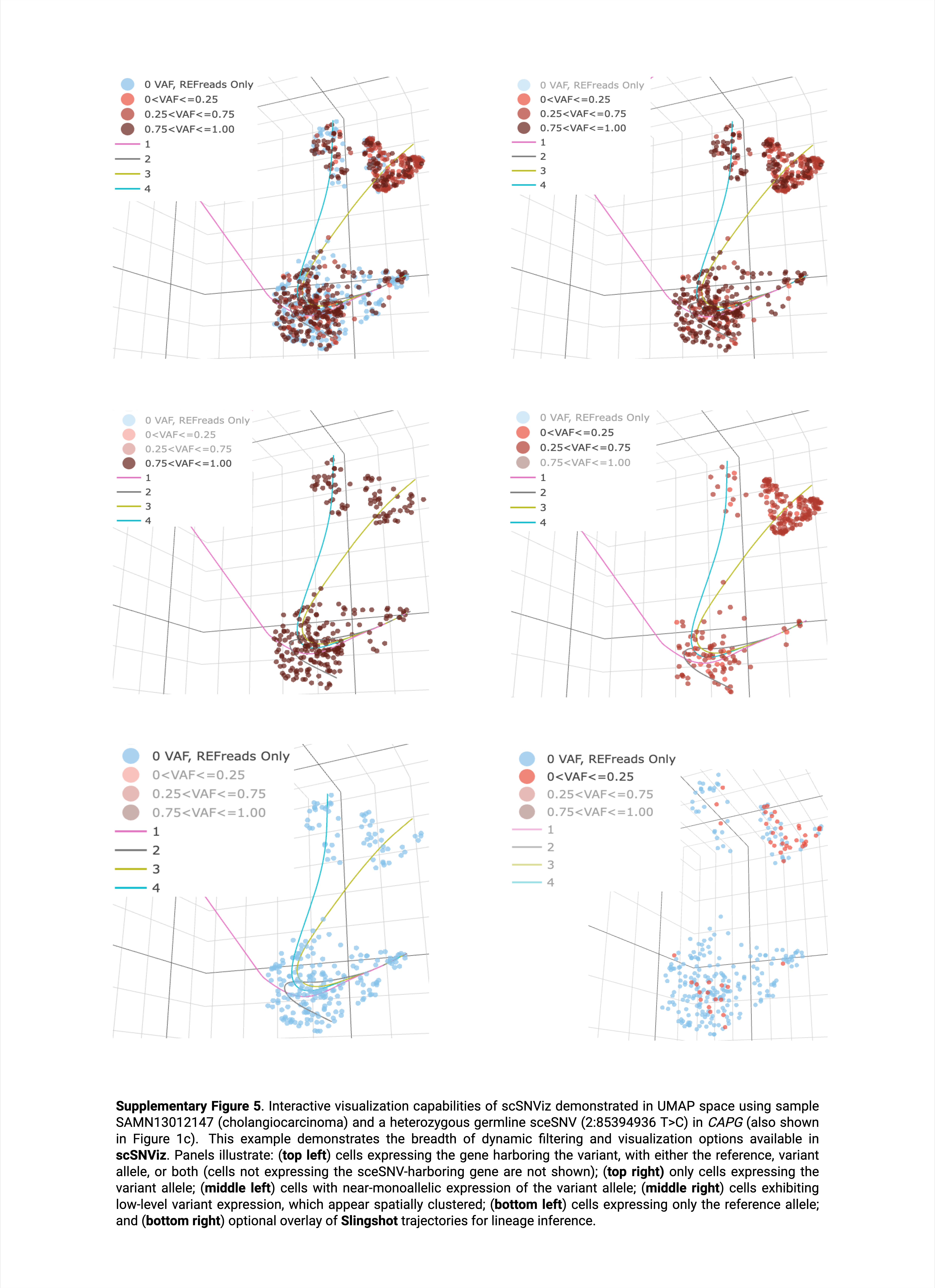

Supplement: btag023_Supplementary_Data [file btag023_supplementary_data.zip › ScSNViz_S_Figure_5.jpg]

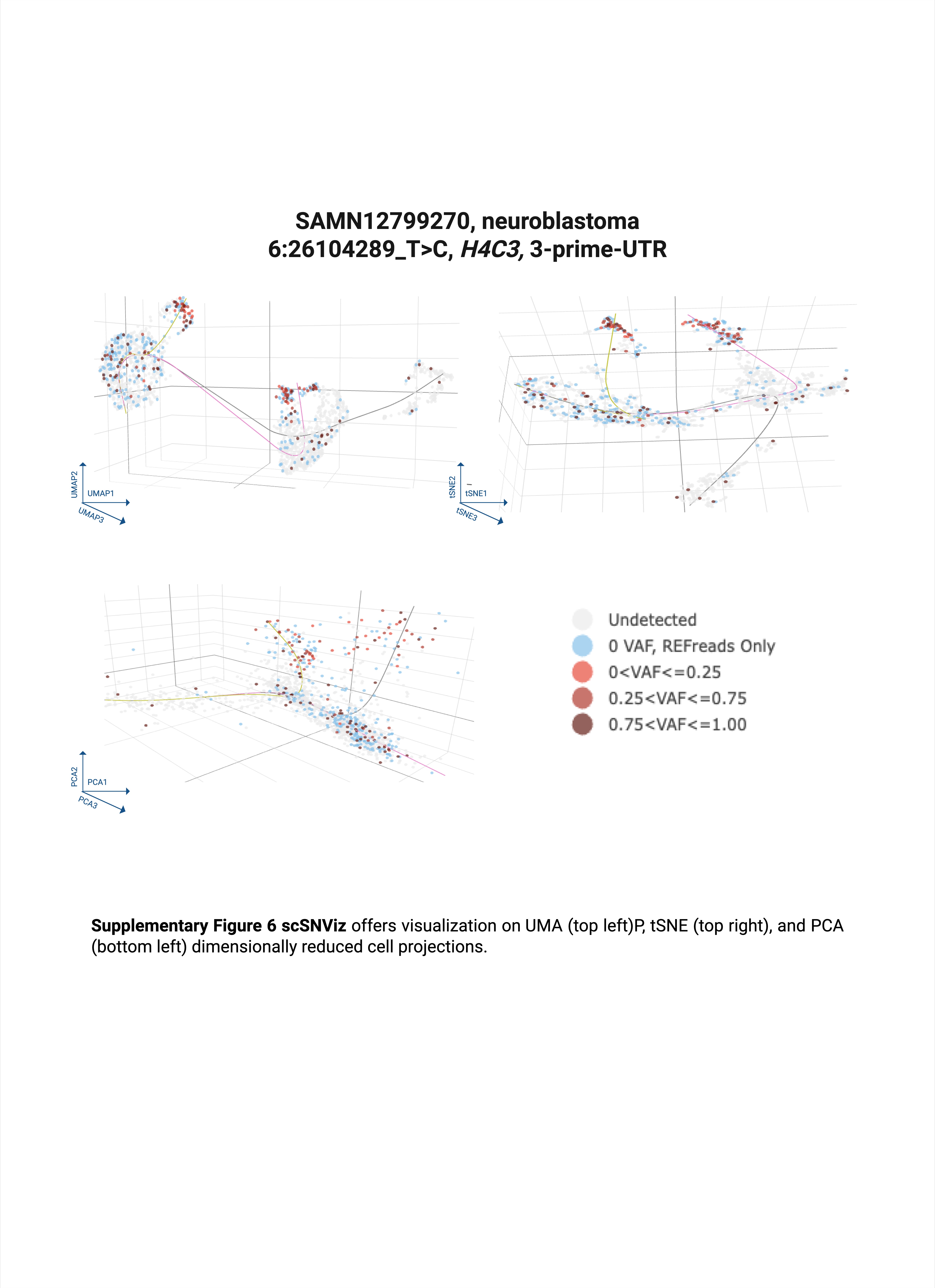

Supplement: btag023_Supplementary_Data [file btag023_supplementary_data.zip › ScSNViz_S_Figure_6.jpg]

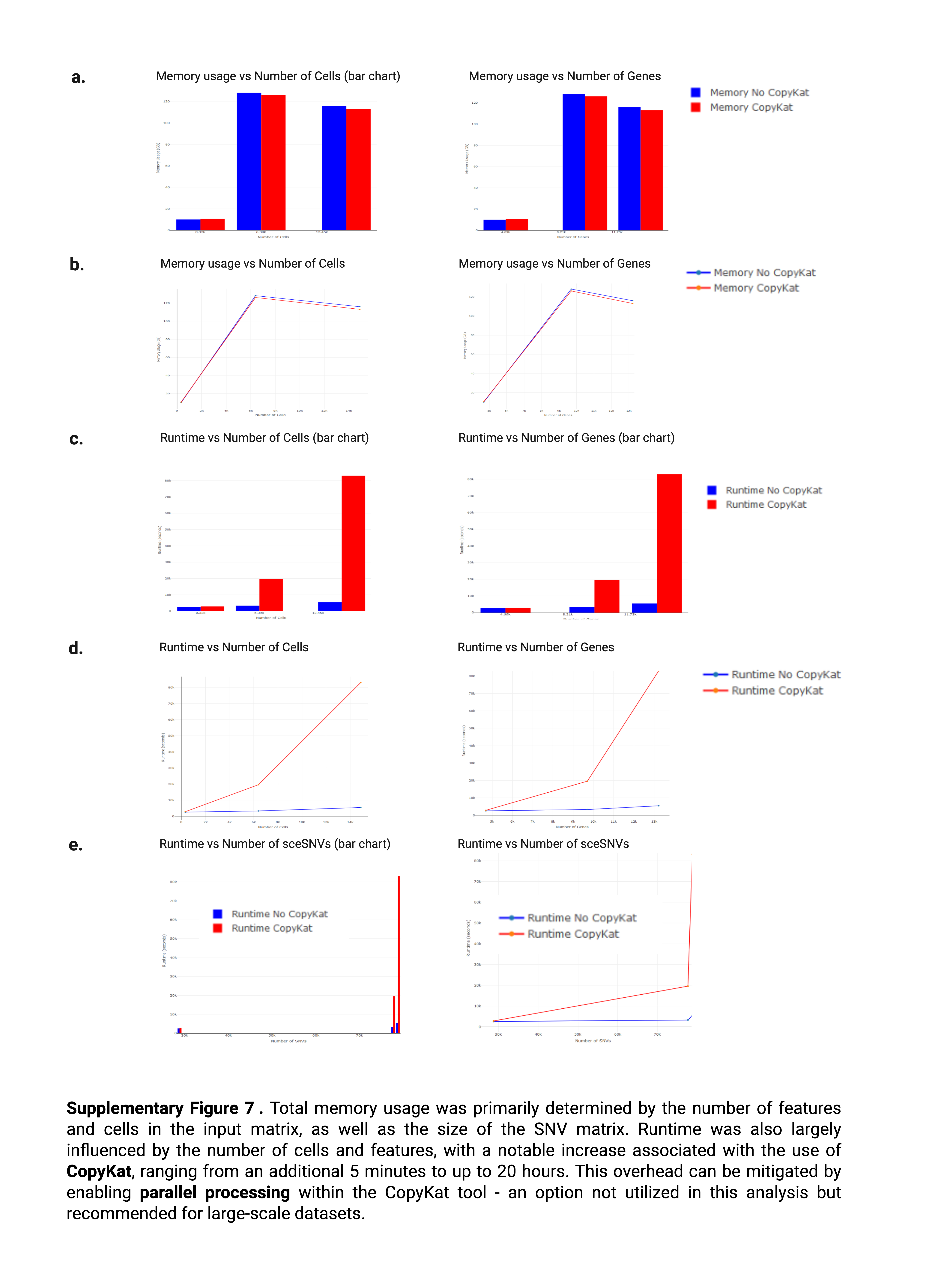

Supplement: btag023_Supplementary_Data [file btag023_supplementary_data.zip › ScSNViz_S_Figure_7.jpg]

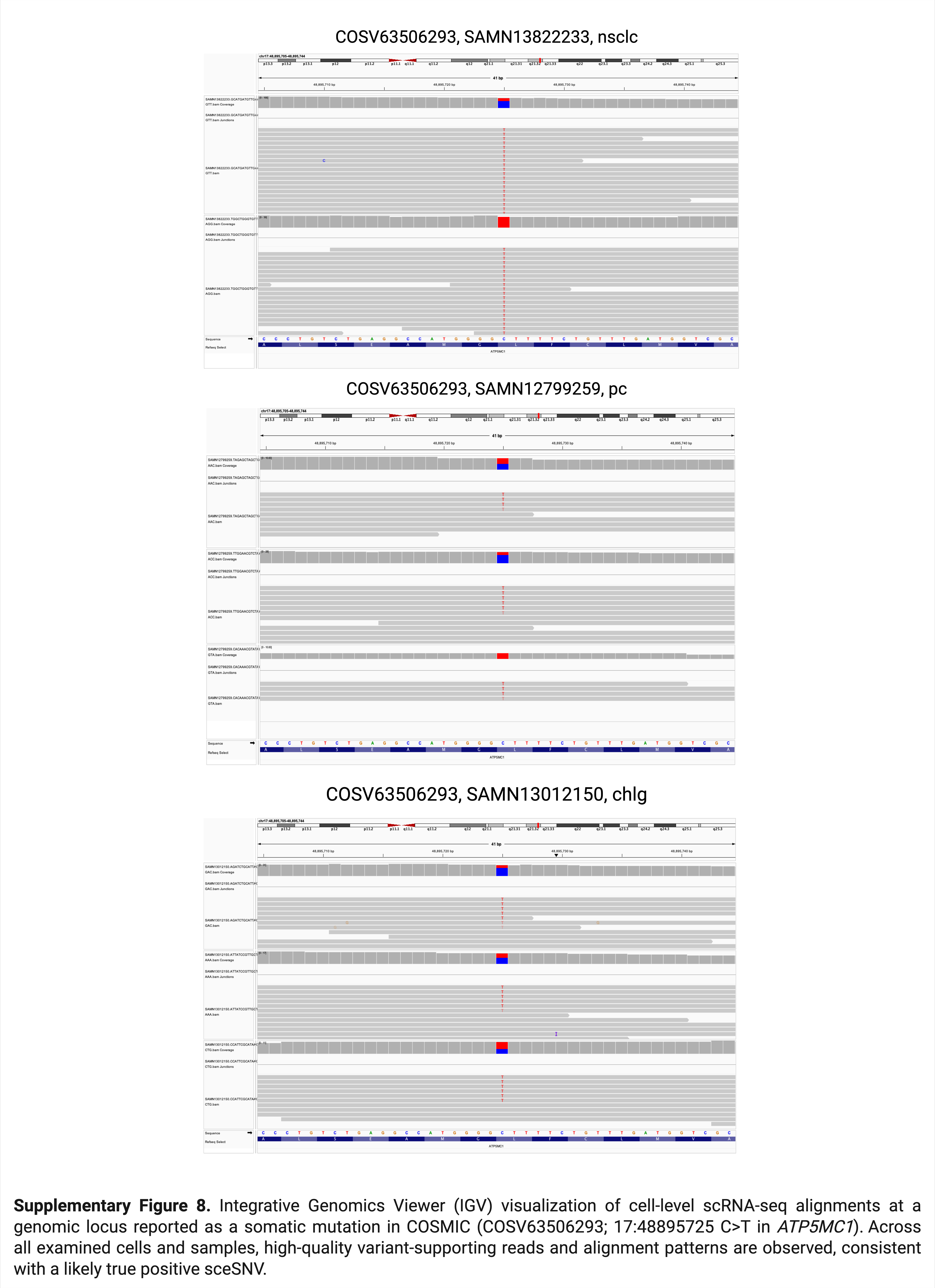

Supplement: btag023_Supplementary_Data [file btag023_supplementary_data.zip › ScSNViz_S_Figure_8.jpg]

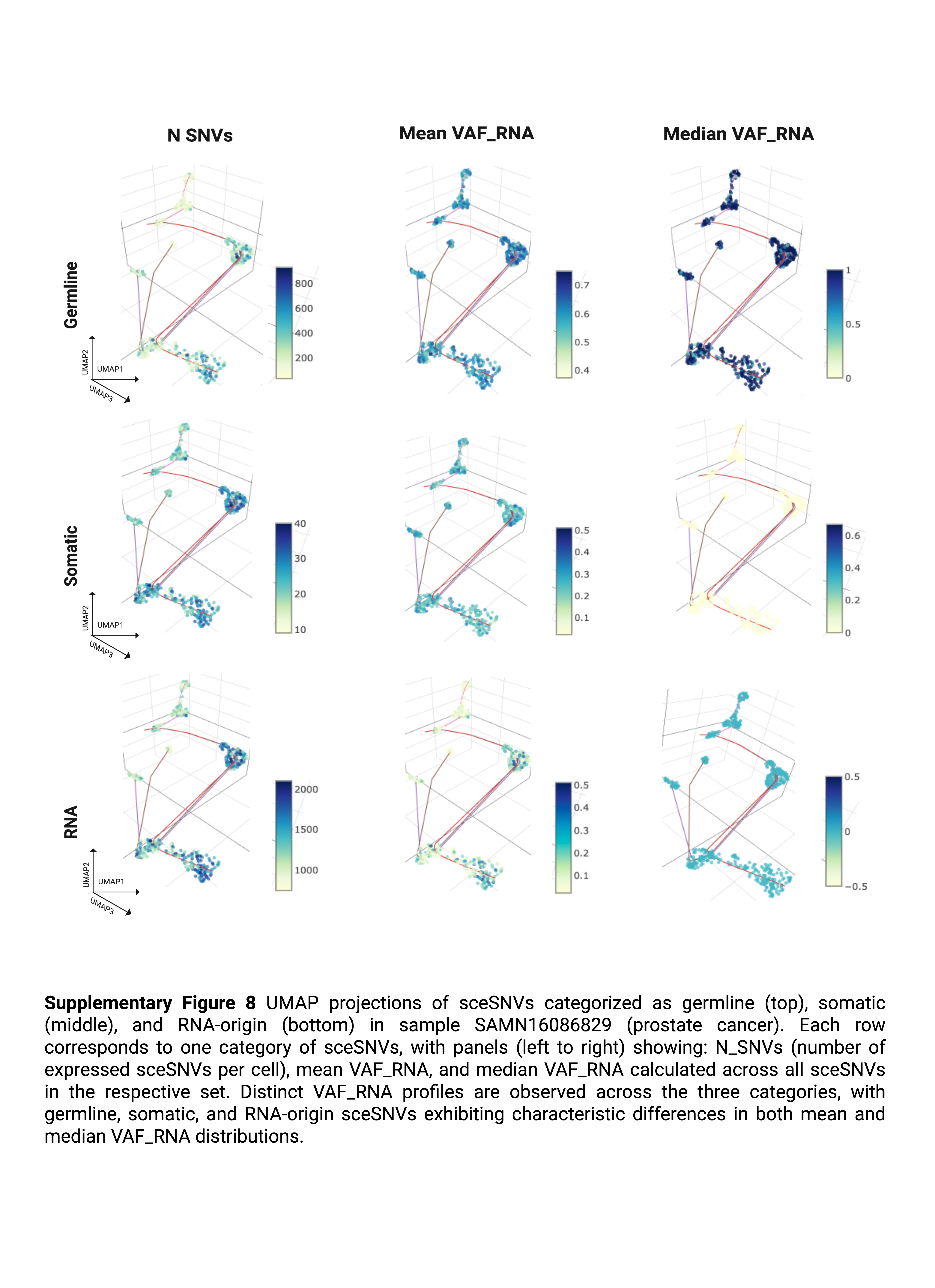

Supplement: btag023_Supplementary_Data [file btag023_supplementary_data.zip › ScSNViz_S_Figure_9.jpg]
